# Supplementary material for: Hepatitis C antibody screening and determinants of initial and duplicate screening in the baby boomer patients of six urban primary care clinics
Source: PLoS One. 2020 Jul 9;15(7):e0235778. doi: 10.1371/journal.pone.0235778 (PMC7347094; doi:10.1371/journal.pone.0235778)
Supplement: Attachment — Submitted filename: Response to Reviewers.pdf [file pone.0235778.s002.pdf]

Jefferson Health.

Department of Medicine
Division of Infectious Diseases

1015 Chestnut Street, Suite 1020
Philadelphia, PA 19107
T 215-955-7785
F 215-955-9362

Nana Aburjania, MD
Katherine A. Belden, MD
Caroline C. Brugger, CRNP
Rachel Carr, PA-C
Dagan Coppock, MD
Joseph A. DeSimone, MD
Phyllis Flomenberg, MD
Elizabeth Gancher, MD
Sarah E. Green, CRNP-BC
Molly Hancock, PA-C
Bryan Hess, MD
Carolyn Kramer, MD
Tricia Royer, DO
Lisa A. Spacek, MD, PhD
Kathleen Squires, MD
Gustavo Vasquez, MD
Devin Weber, MD
John Zurlo, MD

May 12, 2020

To the Editors of PLoS ONE:

We are resubmitting the manuscript previously titled "Screening Baby Boomers for Hepatitis C in Urban Primary Care Clinics: Does Duplicate Testing Make a Difference?" for further review. Reviewer comments have been considered and the paper has been edited to reflect the recommended revisions. We have itemized the comments and our responses to those comments below.

Editor comments:

Concern: "the aim of the study looks confusing. Reading the manuscript title, I expected that duplicate testing is a new strategy for screening of Hep C patients and the study is going to evaluate this strategy."

Response: We have changed the title to better reflect the aims of the study.

Concern: "Reading introduction and aim, I thought the duplicate testing is something unexpected in the primary care clinics but it is not elucidated why it cannot be prevented while they have EHR to document the HCV testing."

Response: The study's introduction and study aims have been re-written to clarify the goals of the paper. Previously described data regarding duplicate testing in the setting of available EHR records has been cited.

Concern: "In Data Availability, authors included "Yes - all data are fully available without restriction". Is there any dataset they want to share with the readers?"

Response: We will be sharing our dataset with readers with this submission.

Concern: "The Manuscript Title, PICO, aims, results and discussion should be synchronized to present the work done by the authors. As I included above, these are yet confusing, decrease the quality of evidence presentation. In introduction, there is little about what is the burden (economic, etc.) of HCV duplicate testing in a population with little ongoing risky behaviors. Why the duplicate testing is important? is it a problem? how can it impact the HCV screening? The aim should be expressed clearly considering the work done and the results will be presented."

Response: The study's introduction has been re-written to clarify the aims of the study. As the study was not a true interventional study (that is, there is no control group), it has been more correctly framed based on its observational aims. The edited version synchronizes the study's introduction and aims with the paper's results and discussion.

Concern: "In Abstract, "aggressive screening efforts in high prevalence populations have led to significant costs despite a lack of clinical utility". I am very confused with that statement. WHO

recommends screening in populations with high prevalence of HCV (>2% or >5%) such as PWID, FSWs, etc."
Response: The abstract has been re-written to clarify the above as well as the goals of the paper.

Concern: "The methods, patient recruitment and documentation is not clear for the readers. As I get, this is a retrospective study using the data accumulated in EHR. I am not against using such data however, the reader needs to know what exactly happened. Was the screening started before 2012? how the baby boomers linked to diagnostics? what were the diagnostic services (methods, labs, etc)? how the results were documented in EHR? was there any counseling from Labs or clinics after testing?"
Response: The methods section now explains that screening rates were evaluated starting in 2012 as this was the year the new CDC guidelines were implemented. More details were provided regarding the data extraction as structured fields linked to baby boomer data. A statement was added regarding follow-up counseling provided by the C for Cure program.

Concern: "Were All the retestings duplicates? any triplicates?"
Response: There were only three triplicates noted in the study. This has been added to the results section.

Concern: "I am confused why the authors observed the retesting in those with negative HCV Ab as an appropriate phenomenon while these individuals mostly have no risk for transmission of HCV."
Response: This has been clarified in the Definitions sub-section of the Methods section with a citation to the AASLD-IDSA HCV guidelines.

Concern: "What was included in the data of Figure 1? The individuals?, the test results? or the visits in clinic?"
Response: The findings in Figure 1 have been clarified in the Results section.

Concern: "What was the number of individuals? number of tests? number of visits in clinic? these can be included in the results."
Response: The total number of patients and their characteristics are now described in Table 1 and paragraph 1 of the results section. The total number of tests performed is now included in the results section (line 117). We do not have the total number of clinic visits available.

Concern: "A Table presenting baseline characteristics of patients is needed."
Response: This table has been created and is now Table 1.

Concern: "In Figure 2, it is not clear what the duplicate tested branch is presenting? the initial testing results or the retesting results."
Response: The branchpoint has now been clarified that the results are of the retesting (duplicate) results.

Concern: "In Table 1, I guess public insurance should have OR more than 1."
Response: In the univariate analysis predicting ever tested (as shown in what was table 1 but is now table 2) with the same set of subjects as in the multivariate, public insurance does indeed have an OR greater than 1 (1.084) as would be expected since the percentage ever tested is

greater in the public group than the private group. However, the analysis in table 2 is multivariate, adjusted for the other variables, and the direction of the association is reversed by the confounder correction. We redid the analyses with the same variables and coding to be sure there was not an accidental switch such that the wrong group was being counted as the reference in the model, and this is not the case. It appears that this small but statistically significant association is tied to other factors. Adding just race or just practice to the model, in addition to insurance type, reverses the direction of the association for insurance type. Adding sex or birth year high/low does not. So, it appears that the association of insurance type with ever tested is confounded by associations with race and practice, which makes sense.

Concern: "In Table 2, the second column can be used for presentation of HCV tested more than once and the third column for HCV tested once."
Response: We have switched the columns accordingly.

Concern: "95% CI of OR for gender in Table 2?"
Response: The CI of OR for gender has been included.

Reviewer #1:
Concern: The authors provide more exact information regarding eligibility criteria and study type in a specific section in the methods part.
Response: We have clarified the inclusion criteria in the Study Population sub-section. We have added a statement about study design under the new sub-section, Study Design.

Concern: "The authors provide P-value for reported odds ration in the results part."
Response: P-values are included in our tables reporting odds ratios (now Tables 2 and 3).

Reviewer #2:
Concern: "It is important to note that at the current time the recommendation is the screen all adults and all pregnant women, this manuscript should be updated to look at both baby boomer "duplicate screening" and potential problems with "duplicate screening" in all adults and all pregnant women"
Response: This dataset only includes data for baby boomers. We have now included the expansion of our study to all adults.

Concern: "There is a statement in the abstract that there is a lack of clinical utility for screening but this is not correct: utility and cost effectiveness has been proven please see AASLD and CDC guidelines, surveillance is recommended in at risk individuals, yes this is "duplicate" but it follows guidelines since at risk patients must be tested regularly and is not wasteful
There is a clear need for "duplicate testing" or "repeat testing" in at risk individuals who show evidence of high-risk behavior, How did the authors document that there was unnecessary duplicate testing, is there adequate documentation in the EMR that these are low risk individuals with no high-risk behavior?"
Response: In our limitations section we now address that our study is limited due to lack of knowledge regarding comorbidities that might increase risk. We have labeled patients who have had repeat antibody screening if their first screen is negative as "potentially appropriate" to address this concept within the given limitations of our data.

Concern: "AST and ALT are liver enzymes and or not liver function tests, were liver function test assessed or liver enzymes assessed?"
Response: We did not collect liver function tests as a component of our dataset. However, this is an excellent recommendation for data to be collected in future studies.

Reviewer #3
Concern: "All the analyses should be adjusted for the cluster effect, as there are clusters (clinics) in the data.
Response: Adjusting for clusters in the multivariate analysis is a reasonable suggestion, but we do not believe it quite applies here. If we had randomly selected 5 units out of 20 and wanted to generalize to all 20, we would have to take into account the fact that the 5 might be atypical in some way. For example, if half the 20 units had a positive association between male gender and testing, while the other half had a negative association, we could easily by chance select 5 that showed the positive association, a different 5 that showed the negative, or 5 that were so mixed as to show no association. Adjustment for clustering would address that risk and give more generalizable results.

However, in this study we used essentially all of the units within the system. Our practices are not a sample from a larger citywide or nationwide set. They are a complete set within a single institution. Adjusting for clustering would not help with generalization to the system, since we included all the units (and all relevant patients within each). There is a question of generalization to the broader world, but that is not easily addressed given that this was a single institution study. If we had just taken one large unit from this study and analyzed that, there would be no cluster effect that we could control for, but exactly the same question of generalizability.

So, we believe that our analysis, with practices controlled as a fixed factor, and with recognition that the results of a single institutional study may have limited generalizability if there are important differences between systems, is an acceptable approach.

Concern: "A more rigorous conclusion is needed. The conclusion does not support the results. I see that part of duplicate testings were helpful. So, the authors should discuss if the rate of "unnecessary" duplicate testing is practically important."
Response: We edited the conclusion to more strongly follow the results. We clarified that providers concerned about ongoing risk in patients who might have been treated or spontaneously cleared might have evaluated that risk more appropriately through viral load testing.

Additional Requirements
Request: "When submitting your revision, we need you to address these additional requirements: Please ensure that your manuscript meets PLOS ONE's style requirements, including those for file naming. The PLOS ONE style templates can be found at
http://www.plosone.org/attachments/PLOSOne_formatting_sample_main_body.pdf and
http://www.plosone.org/attachments/PLOSOne_formatting_sample_title_authors_affiliations.pdf"
Response: We have reviewed the guidelines and have re-formatted our paper accordingly.

Request: "Please amend your current ethics statement to include the full name of the ethics committee/institutional review board(s) that approved your specific study and confirm that your

named institutional review board or ethics committee specifically approved this study.
Once you have amended this/these statement(s) in the Methods section of the manuscript, please add the same text to the "Ethics Statement" field of the submission form (via "Edit Submission")."

Response: We have made the changes as requested.

Request: "Please include your tables as part of your main manuscript and remove the individual files. Please note that supplementary tables (should remain/ be uploaded) as separate "supporting information" files"

Response: We have added our tables and figures to the manuscript.

Thank you for your consideration of this re-submission. We hope these revisions help to address your concerns. Please contact me with any further questions.

Sincerely,

Dagan Coppock, MD

Dagan Coppock, MD Thomas Jefferson University
Division of Infectious Diseases
(c) 203-915-7302
(o) 267-503-8845
(f) 215-503-3417
dagan.coppock@jefferson.edu
