# Supplementary material for: Hepatitis C antibody screening and determinants of initial and duplicate screening in the baby boomer patients of six urban primary care clinics
Source: PLoS One. 2020 Jul 9;15(7):e0235778. doi: 10.1371/journal.pone.0235778 (PMC7347094; doi:10.1371/journal.pone.0235778)
Supplement: Attachment — Submitted filename: Response to Reviewers.pdf [file pone.0235778.s003.pdf]

Jefferson Health.

Department of Medicine
Division of Infectious Diseases

1015 Chestnut Street, Suite 1020
Philadelphia, PA 19107
T 215-955-7785
F 215-955-9362

Nana Aburjania, MD
Katherine A. Belden, MD
Caroline C. Brugger, CRNP
Rachel Carr, PA-C
Dagan Coppock, MD
Joseph A. DeSimone, MD
Phyllis Flomenberg, MD
Elizabeth Gancher, MD
Sarah E. Green, CRNP-BC
Molly Hancock, PA-C
Bryan Hess, MD
Carolyn Kramer, MD
Tricia Royer, DO
Lisa A. Spacek, MD, PhD
Kathleen Squires, MD
Gustavo Vasquez, MD
Devin Weber, MD
John Zurlo, MD

June 20, 2020

To the Editors of PLoS ONE:

We are resubmitting manuscript PONE-D-20-03702R1, titled "Hepatitis C antibody screening and determinants of initial and duplicate screening in the baby boomer patients of six urban primary care clinics," for further review. Reviewer comments have been considered and the paper has been edited to reflect the recommended revisions. We have itemized the comments and our responses to those comments below.

Reviewer #1:

Comment: "I checked the revised version of the manuscript together with authors' comments. All of my concerns have been resolved."

Response: We thank you for your comments.

Reviewer #2:

Comment: "In the discussion, a small expansion/discussion of the limits of the study due to limited data on risk behaviour or co-morbidities that could lead to repeat testing (not duplicate testing {that implies that the second and third test were not necessary})."

Response: Thank you for the recommendation regarding the limitations. In the discussion section, we have expanded on the limits of the study due to our limited dataset. Further, your comments raise excellent points about our terminology. We have changed the language throughout the paper to clarify that duplicate testing may not be "unnecessary," particularly in the context of ongoing risk/co-morbidities. We have elucidated that repeat antibody screening in patients who already have a baseline positive antibody screen is an inappropriate means for assessing patients with ongoing risk and that viral load testing would be more appropriate.

Reviewer #3:

Comment: "for the concern I raised about cluster effect, further attention is needed. In studies where the data is grouped in the form of clusters (here, the clinics), the within-cluster correlations will shrink the total confidence intervals. So, the authors need to correct the confidence-intervals for the "intra-class correlation coefficient". Including all the clinics in your analysis, will not correct the "cluster/design effect", as this issue deals with the correlations that exist within your clusters (i.e., clinics)."

Response: This was a very helpful recommendation. We re-performed our regressions, controlling for clustering by clinic. Our odds rations changed and our confidence intervals widened with a decrease in significance for specific variables. We have replaced our previous regression results with the clustered results and have changed our discussion accordingly.

Thank you for your consideration of this re-submission. We hope these revisions help to address your concerns. Please contact me with any further questions.

Sincerely,

Dagan Coppock, MD

Dagan Coppock, MD
Thomas Jefferson University
Division of Infectious Diseases
(c) 203-915-7302
(o) 267-503-8845
(f) 215-503-3417
dagan.coppock@jefferson.edu
